# Correction: A composite lateral flow test substrate by capillary deposition of cellulose on synthetic paper

**DOI:** 10.1039/d6ra90067d

**Published:** 2026-07-06

**Authors:** Qinghao He, Jiahua Zhong, Haonan Li, Xionghui Li, Yixi Shi, Muyang Zhang, Jie Zhou, Hao Chen, Xinyi Chen, Zhuoting Han, Lok Ting Chu, Huiru Zhang, Weijin Guo

**Affiliations:** a Department of Biomedical Engineering, Shantou University Shantou 515063 Guangdong China guoweijin@stu.edu.cn; b Department of Laboratory Medicine, The Second Affiliated Hospital of Guangdong Medical University Zhanjiang 524003 China; c Institute of Biochemistry and Molecular Biology, Guangdong Medical University Zhanjiang 524023 Guangdong China; d Neusoft Institute Guangdong Foshan 528225 Guangdong China zhanghuirugd@foxmail.com

## Abstract

Correction for ‘A composite lateral flow test substrate by capillary deposition of cellulose on synthetic paper’ by Qinghao He *et al.*, *RSC Adv.*, 2026, **16**, 29940–29949, https://doi.org/10.1039/D6RA01317A.

The authors regret that an incorrect version of Fig. 7 was included in the original article. The correct version of Fig. 7 is presented below.

**Fig. 1:**
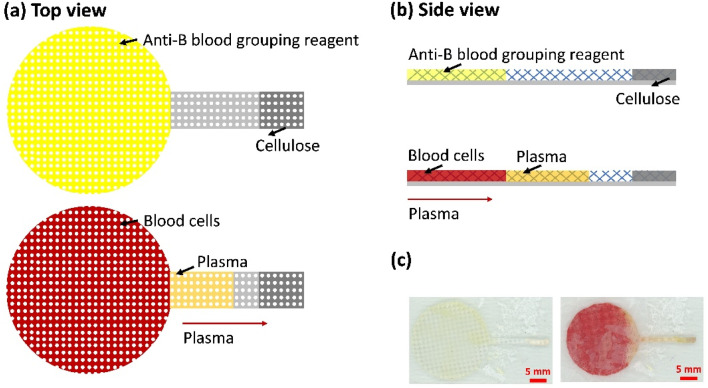
Separation of plasma from whole blood on a test strip by the composite substrate. (a) Schematic top view of experimental procedures of plasma separation. The upper picture of (a) shows a test strip after coating agglutinating antibody on the sample area. The lower picture of (a) shows the plasma separation and collection at the end of plasma channel. (b) Schematic side view of experimental procedures of plasma separation. (c) Pictures of a test strip before and after adding whole blood samples.

The Royal Society of Chemistry apologises for these errors and any consequent inconvenience to authors and readers.

